# Prospective Analysis of Cutaneous Adverse Drug Reactions Encountered in a Tertiary Care Hospital

**DOI:** 10.7759/cureus.65284

**Published:** 2024-07-24

**Authors:** Pravin Dhage, Smita Mali, Sudhir Pawar, Bakul Naik, Varsha Mali

**Affiliations:** 1 Pharmacology and Therapeutics, Lokmanya Tilak Municipal Medical College and General Hospital, Mumbai, IND

**Keywords:** drug eruptions, severe cutaneous reactions, morphological pattern, self-medication, pharmacovigilance, cutaneous adverse drug reactions

## Abstract

Introduction: Adverse drug reactions (ADRs), including cutaneous adverse drug reactions (CADRs), are significant global health concerns with India among the top affected countries. CADRs represent a significant concern in healthcare, impacting the skin, its appendages and mucous membranes ranging from mild rashes to severe, life-threatening conditions like Stevens-Johnson syndrome and toxic epidermal necrolysis. Self-medication with over-the-counter drugs is a notable public health issue linked to CADRs. Clinical trials often miss long-term and rare CADRs making early detection and monitoring crucial. This study aims to evaluate CADRs by assessing their causality, severity and preventability; determining onset lag time; identifying morphological patterns; and investigating associations with different drug classes. It also explores the links between self-medication and CADRs and analyses related outcomes. This research addresses gaps in understanding CADRs' epidemiology, impact and management providing valuable insights for healthcare practitioners.

Material and methods: A 12-month prospective observational study conducted at a tertiary care hospital involved dermatology patients from both outpatient and inpatient units. Inclusion criteria comprised patients diagnosed with CADRs by physicians in the outpatient department (OPD) (active surveillance) and reported cases to pharmacovigilance unit (passive surveillance) while those unwilling to provide written consent were excluded.

Result: The majority (44.25%) of the patients were aged 18-39 years. Maculopapular rash (53.98%) and urticarial rash (9.73%) were the most common CADR types. Anti-bacterials (42.63%) were the primary suspected drug class. Serious CADRs were predominant (74.34%) with 1.77% resulting in fatalities. Severity was moderate in 79.65% and mild in 17.7% of the cases. Preventability was low (5.31%) with three CADRs attributed to self-medication. Recovery was seen in 46.9% of the patients with 42.48% still in recovery at discharge and a mortality rate of 1.77% due to Stevens-Johnson syndrome.

Conclusion: A comprehensive pharmacovigilance system for continuous monitoring of patients' health status can lead to opportunities to reduce the CADRs, lower drug-related morbidity and rationalize drug therapy.

## Introduction

The World Health Organization (WHO) defines an adverse drug reaction (ADR) as "a response to a drug that is noxious and unintended and occurs at doses used in man for prophylaxis, diagnosis or therapy of a disease or for modification of physiological function" [1]. This definition specifically excludes poisoning and unintended but beneficial effects, focusing on harmful reactions in relation to drug use. Skin represents a primary target organ for ADRs.

A cutaneous adverse drug reaction (CADR) denotes an undesirable alteration in the structure or function of the skin, its appendages or mucous membranes encompassing all adverse events related to drug eruption irrespective of their underlying causes [2]. The incidence of dermatological ADRs among inpatients in developed countries ranges from 1% to 3% [3,4], while in developing nations like India, it ranges from 2% to 5% [5]. ADRs are significant contributors to mortality globally with India ranking among the top 10 countries affected [6]. Among dermatological conditions, drug eruptions rank as one of the most common [2] and these reactions span a broad spectrum, ranging from transient maculopapular rashes to severe conditions like toxic epidermal necrolysis (TEN) [7]. Common skin drug eruptions often manifest as pruritus, maculopapular eruptions, urticaria, angioedema, phototoxic and photoallergic reactions, fixed drug reactions, vesiculobullous reactions and exfoliative lesions resembling allergic responses and are categorized as drug hypersensitivity reactions [5].

Severe cutaneous adverse reactions (SCARs) that can endanger a patient's life include Stevens-Johnson syndrome (SJS), toxic epidermal necrolysis (TEN), drug reaction with eosinophilia and systemic symptoms (DRESS) and acute generalized exanthematous pustulosis (AGEP) [8,9]. The term "severe" is often used to describe the intensity (severity) of a specific event (as in mild, moderate or severe); the event itself, however, may be of relatively minor medical significance (such as severe headache). This is not the same as "serious," which is based on patient/event outcome or action criteria usually associated with events that pose a threat to a patient's life or functioning [1]. Approximately 5%-8% of all hospitalizations worldwide are due to ADRs and CADRs are commonest (30%-45%) among them, responsible for about 2% of hospital admissions [10]. Of these cases, about 2%-7% may be severe with approximately one in 1,000 hospitalized patients experiencing a SCAR [11,12].

Self-medication including the use of over-the-counter (OTC) drugs and old prescriptions without current physician approval is associated with CADRs and poses a public health concern [13]. Establishing the association between CADRs and self-medication can help in public health awareness. The pattern of CADRs changes with new medications and prescription practices highlighting the importance of understanding their precise nature to identify the offending drug [14].

Clinical trials conducted in controlled conditions for a short duration do not provide a complete picture of the long-term and rare ADRs; only about 50% of drug reactions can be detected in premarketing trials [15]. Hence early detection, evaluation and monitoring of ADRs particularly severe CADRs are crucial for patient safety to prevent morbidity and mortality.

Therefore, this study was conducted with aim to evaluate CADRs, which includes assessment of the causality, severity, seriousness, preventability, outcomes and morphological pattern of CADRs, investigating the onset lag time, associations with specific drug classes and the link between self-medication with CADRs.

## Materials and methods

This is a single-center, prospective, observational study conducted in a tertiary care hospital (March 2021 to February 2022). Evaluation of the diagnosed CADR patients was done by the active surveillance (patients in outpatient department (OPD) and ward) and passive surveillance (cases reported to the pharmacovigilance cell of the hospital). Then the patients were enrolled according to the inclusion criteria, i.e., provisionally diagnosed CADR patients by the physician in dermatology OPD and ward and those reported to the pharmacovigilance cell. The patient, or their guardian or legally accepted representative (LAR), not willing to give written informed consent were excluded from the study.

After obtaining the written informed consent, CADRs were identified by patient interviews, detailed history of drug intake and case record reviews. All the patients were monitored until the CADR is recovered or till the date of discharge. All the different CADRs were classified according to the Medical Dictionary for Regulatory Activities (MedDRA) system organ classification. The above-mentioned data was recorded in a Case Record Form, which was subsequently entered in Microsoft Excel (Microsoft Office Professional Plus 2021, Version 2406, Microsoft, Redmond, WA) and analyzed.

## Results

Demographic data

**Figure 1:**
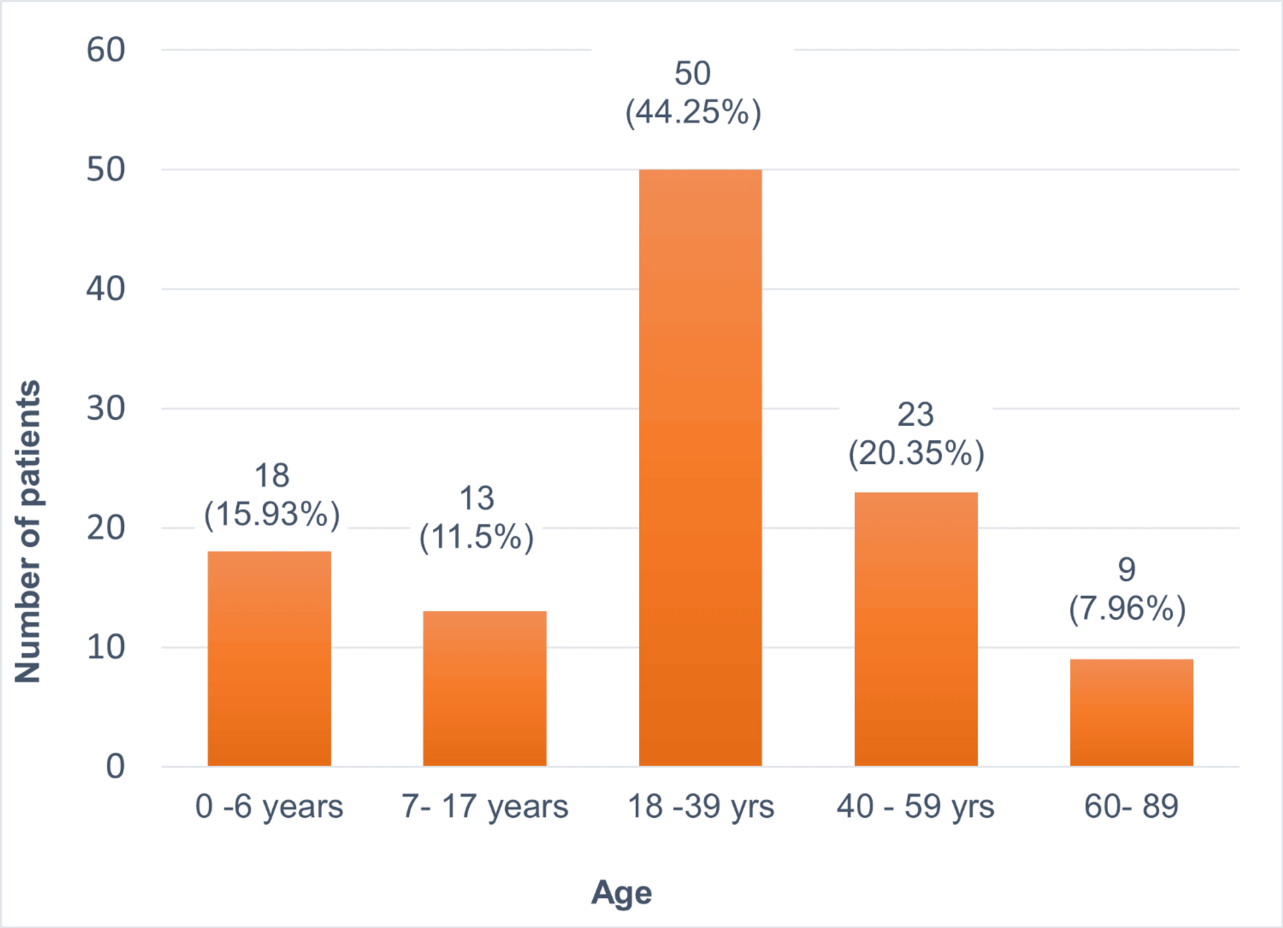
Age-wise distribution of patients experiencing cutaneous adverse drug reactions (n=113)

Among 113 patients diagnosed with CADRs within the dermatology outpatient and inpatient department, males comprised 59.29% (n=67) and females 40.71% (n=46), resulting in a male-to-female ratio of 1.46:1. Out of 113 patients, 18 (15.93%) were aged 0-6 years, 13 (11.5%) aged 7-17 years, 50 (44.25%) aged 18-39 years, 23 (20.35%) aged 40-59 years and nine (7.96%) aged 60-89 years with the youngest patient being three months old and the oldest 86 years old (Figure 1). The mean age of patients experiencing CADRs in dermatology OPD and wards was 30.46 ± 19.67 SD years with a median age 29 years (13.5-45). Also, six patients (5.31%) had a prior history of CADRs.

Analysis of CADRs as per the morphological pattern

According to MedDRA system organ classification (MeDRA SOC), the majority of the reactions were categorized under skin and subcutaneous tissue disorders (65 CADRs, 57.52%) followed by skin and subcutaneous tissue disorders, immune system disorders (41 CADRs, 36.28%) and skin and subcutaneous tissue disorders, general disorder and administration site condition (seven CADRs, 6.2%). The most common CADR was maculopapular rash (61 patients, 53.98%), urticarial rash (11 patients, 9.73%) followed by SJS (10 patients, 8.86%). Other common CADRs were Redman syndrome (six patients, 5.31%) and fixed drug eruption (six patients, 5.31%) (Table 1).

**Table 1:** Morphological pattern of cutaneous adverse drug reactions according to MedDRA system organ class (n=113) SOC: system organ class; DRESS: drug reaction with eosinophilia and systemic symptoms.

MedDRA SOC	CADRs	Frequency
Skin and subcutaneous tissue disorders (57.52%)	Maculopapular rash	61 (53.98 %)
	Itching	2 (1.77%)
	Redness	1 (0.88%)
	Blisters	1 (0.88%)
Skin and subcutaneous tissue disorders, immune system disorders (36.28%)	Urticarial rash	11 (9.73%)
	Steven-Johnson syndrome	10 (8.86%)
	DRESS	6 (5.31%)
	Redman syndrome	6 (5.31%)
	Toxic epidermal necrolysis	3 (2.65%)
	Erythema multiforme	3 (2.65%)
	Acute urticaria	2 (1.77%)
Skin and subcutaneous tissue disorders, immune system disorders, general disorder and administration site condition (6.2%)	Fixed drug eruption	6 (5.31%)
	Lichenoid drug eruption	1 (0.88%)

Suspected drugs for these CADRs

This study examined 190 drugs for their potential to cause CADRs in 113 confirmed cases. Among these, single drugs were implicated in 71 cases while combinations of two different drugs were suspected in 23 cases and multiple drugs were involved in 19 cases. As mentioned in Table 2, the most commonly suspected drug classes in causing CADRs were anti-bacterials (42.63%), antitubercular drugs (14.21%), antiepileptic drugs (11.58%) and aantipyretic drugs (4.74%). Out of the 113 patients, suspected drugs were withdrawn in 97, continued in 12 and course of treatment completed in four patients during the study.

**Table 2:** Suspected drug and its classes causing CADRs (n= 190). NSAID: Non-steroidal anti-inflammatory drugs.

S. No.	Drug class	Frequency	Percentage (%)	Drugs
1	Anti-bacterial	81	42.63	Linezolid
				Amoxicillin
				Amoxicillin + Clavulanic acid
				Metronidazole
				Norfloxacin
				Ciprofloxacin
				Vancomycin
				Dapsone
				Doxycycline
				Piperacillin + Tazobactam
				Ceftriaxone
				Ornidazole
				Ofloxacin
				Azithromycin
				Sulfamethoxazole + Trimethoprim
				Levofloxacin
				Meropenem
				Imipenem
				Cilastatin
				Gentamicin
				Streptomycin
				Cefixime
				Teicoplanin
				Piperacillin
				2	Antitubercular	27	14.21	Rifampicin
Ethambutol							
Pyrazinamide							
Isoniazid							
Cycloserine							
Clofazimine							
Bedaquiline							
3	Antiepileptic	22	11.58					Phenytoin
				Phenobarbitone
				Lamotrigine
				Carbamazepine
				4	Antipyretic	9	4.74	Paracetamol
5	Antifungal	8	4.21	Amphotericin
				Fluconazole
				Terbinafine
				Itraconazole
6	Antiviral	7	3.68	Ritonavir
				Stavudine
				Lopinavir
				Dolutegravir
				Lamivudine
				Tenofovir
7	Antihistamine	6	3.16	Levocetirizine
				Hydroxyzine
8	Anti-malarial	5	2.63	Artemether
				Lumefantrine
				Artesunate
				Hydroxychloroquine
9	Immunosuppressants	4	2.1	Methotrexate
				Sulfasalazine
10	NSAIDs	3	1.58	Diclofenac
				Aspirin
11	Antiemetic	3	1.58	Ondansetron
				Domperidone
12	Proton pump inhibitor	2	1.05	Pantoprazole
13	Retinoid	2	1.05	Acitretin
14	General anesthetic	2	1.05	Propofol
				Ketamine
15	Anti-diabetic	1	0.53	Lente Insulin
16	Anticholinergic	1	0.53	Dicyclomine
17	Anticoagulant	1	0.53	Rivaroxaban
18	Anthelmintic	1	0.53	Albendazole
19	Antispasmodic	1	0.53	Drotaverine
20	Glucocorticoid	1	0.53	Hydrocortisone
21	Vitamin	1	0.53	Pyridoxine
22	Other	2	1.05	Acamprosate
				Atiplate

Among the antibacterial drugs, penicillin and sulfones were most commonly suspected of causing CADRs (16 cases each, 19.75%), followed by glycopeptides (13 cases, 16.05%) and fluoroquinolones (12 cases, 14.81%) (Table 3). Vancomycin was the most common individual antibiotic suspected (12 cases, 6.32%) followed by amoxicillin + clavulanic acid (10 cases, 5.26%) and sulfamethoxazole + trimethoprim (nine cases, 4.74%).

**Table 3:** Antibacterial drugs suspected of causing CADRs (n = 81)

S. No.	Drug class	Class frequency	Percentage (%)	Drug	
1	Penicillin	16	19.75	Amoxicillin + Clavulanic acid	
				Piperacillin + Tazobactam	
2	Sulfone	16	19.75	Sulfamethoxazole + Trimethoprim	
				Dapsone	
3	Glycopeptide	13	16.05	Vancomycin	
				Teicoplanin	
4	Fluoroquinolone	12	14.81	Ofloxacin	
				Levofloxacin	
				Ciprofloxacin	
				Norfloxacin	
5	Cephalosporin	8	9.88	Ceftriaxone	
				Cefixime	
6	Nitroimidazole	4	4.94	Metronidazole	
				Ornidazole	
7	Aminoglycoside	3	3.7	Gentamicin	
				Streptomycin	
8	Macrolide	2	2.47	Azithromycin	
9	Tetracycline	2	2.47	Doxycycline	
10	Carbapenem	2	2.47	Meropenem	
				Imipenem	
11	Oxazolidinone	2	2.47	Linezolid	

Severe cutaneous adverse reactions (SCARs)

Out of the 113 CADRs, 19 SCARs (16.81%) were observed in this study as mentioned in Table 4. The most common SCARs observed are SJS (10 cases, 8.85%), DRESS (six cases, 5.31%) and TEN (three cases, 2.65%). Sulfa drugs (seven cases) and phenytoin (six cases) were among the top in the list of suspected drugs causing SCARs.

**Table 4:** Severe cutaneous adverse drug reactions and suspected drugs (n = 19) SCARs: Severe cutaneous adverse reactions; DRESS: drug reaction with eosinophilia and systemic symptoms.

S. No.	SCARs	Drug frequency	Percentage (%)
1	Steven-Johnson syndrome (10 cases)	Paracetamol - 2	8.85
		Amoxicillin - 1	
		Azithromycin - 1	
		Sulfamethoxazole - 3	
		Phenytoin - 3	
2	DRESS (6 cases)	Phenytoin - 2	5.31
		Dapsone - 3	
		Sulfasalazine - 1	
		Rivaroxaban - 1	
2	Toxic epidermal necrolysis (3 cases)	Phenobarbitone - 2	2.65
		Phenytoin - 1	

Onset lag time

**Figure 2:**
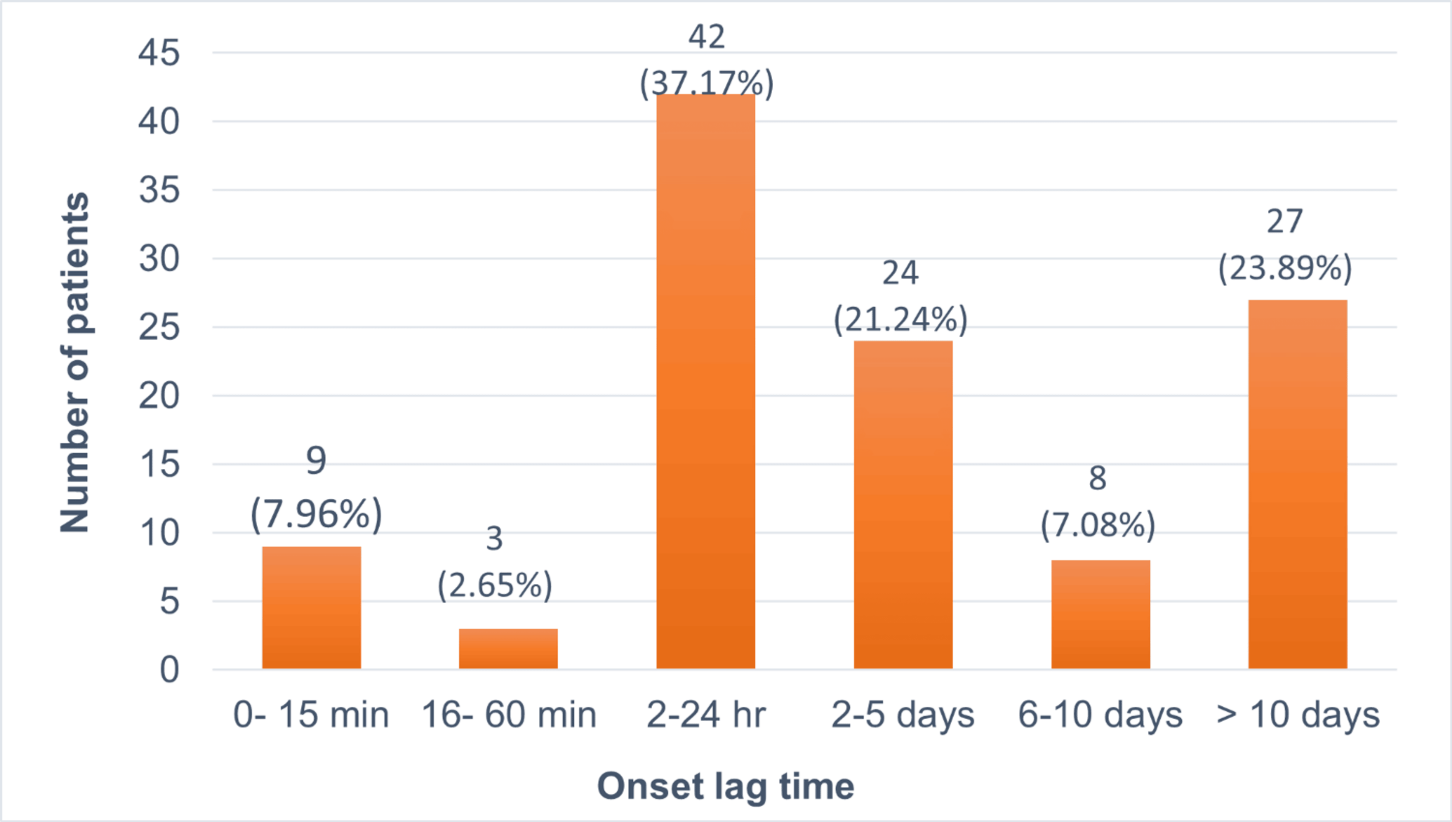
Onset lag time of CADRs in patients (n=113)

Onset lag time can be defined as the time period between the patient ingesting the drug and the appearance of symptoms of CADRs. Out of the 113 patients, the most common onset lag time was between 2 and 24 hours in 42 patients (37.17%). The second most prevalent onset lag time was >10 days in 27 patients (23.89%) followed by two to five days in 24 patients (21.24%). Rapid onset within 0-15 minutes was observed in nine patients (7.96%) while six to 10 days and 16-60 minutes were observed to be the onset lag time in eight patients (7.08%) and three patients (2.65%), respectively (Figure 2).

Causality assessment of CADRs

**Figure 3:**
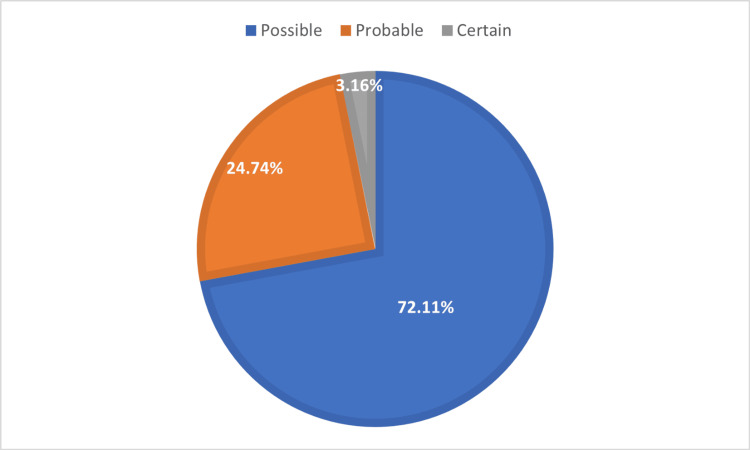
Causality of drugs causing CADRs as per WHO-UMC causality assessment scale. (n=190) WHO-UMC: World Health Organization-Uppasala Monitoring Center.

The causality assessment for all the 190 suspected drugs responsible for CADRs was conducted using the WHO-UMC (World Health Organization-Uppasala Monitoring Center) and Naranjo causality assessment scales. According to the WHO-UMC causality assessment scale, six (3.16%) were certain, 47 (24.74%) were probable and 137 (72.11%) were possible (figure 3). By the Naranjo scale, one (0.53%) was definite and 189 (99.47%) were probable.

Seriousness of the CADRs

The seriousness of CADRs was assessed using the International Conference on Harmonization (ICH) E2A guidelines. A serious adverse event (experience) or reaction is any untoward medical occurrence that at any dose results in death, is life-threatening, requires inpatient hospitalization or prolongation of existing hospitalization, results in persistent or significant disability/incapacity, or a congenital anomaly/birth defect. Out of the 113 cases of CADRs, 29 cases (25.66%) were non-serious while 84 cases (74.34%) were categorized as serious. Among the serious cases, 46 patients (40.71%) required hospitalization or experienced prolonged hospital stays due to the CADR, 35 patients (30.97%) exhibited other medically important reasons for seriousness, two patients (1.77%) had fatal outcomes and one patient experienced a life-threatening CADR (Table 5).

**Table 5:** Seriousness of cutaneous adverse drug reactions (CADRs) (n=113) ADR: Adverse drug reaction.

S. No.	Seriousness criteria	Number of ADRs	Percentage (%)
1	Non-serious	29	25.66
2	Serious	84	74.34
	(a) Hospitalization/prolonged	46 (24+22)	40.71
	(b) Other medically significant	35	30.97
	(c) Death	2	1.77
	(d) Life-threatening	1	0.88
	Total	113	100

Severity of CADRs

In this study, the severity of CADRs was assessed using the modified Hartwig-Siegel scale, Among the 113 CADRs, 20 cases (17.7%) were classified as mild, 90 cases (79.65%) as moderate and three cases (2.65%) as severe.

Preventability of the CADRs

The preventability assessment of CADRs was performed using Modified Schumock-Thornton scale. Six (5.31%) CADRs were preventable and 107 (94.69%) were not preventable.

Cutaneous adverse reactions due to self-medication

Out of the 190 suspected drugs that were associated with CADRs, three drugs - levocetirizine in two different cases and Atiplate (herbal medication) in one case - were taken over the counter. The remaining 187 were prescribed drugs.

Outcome of CADRs

**Figure 4:**
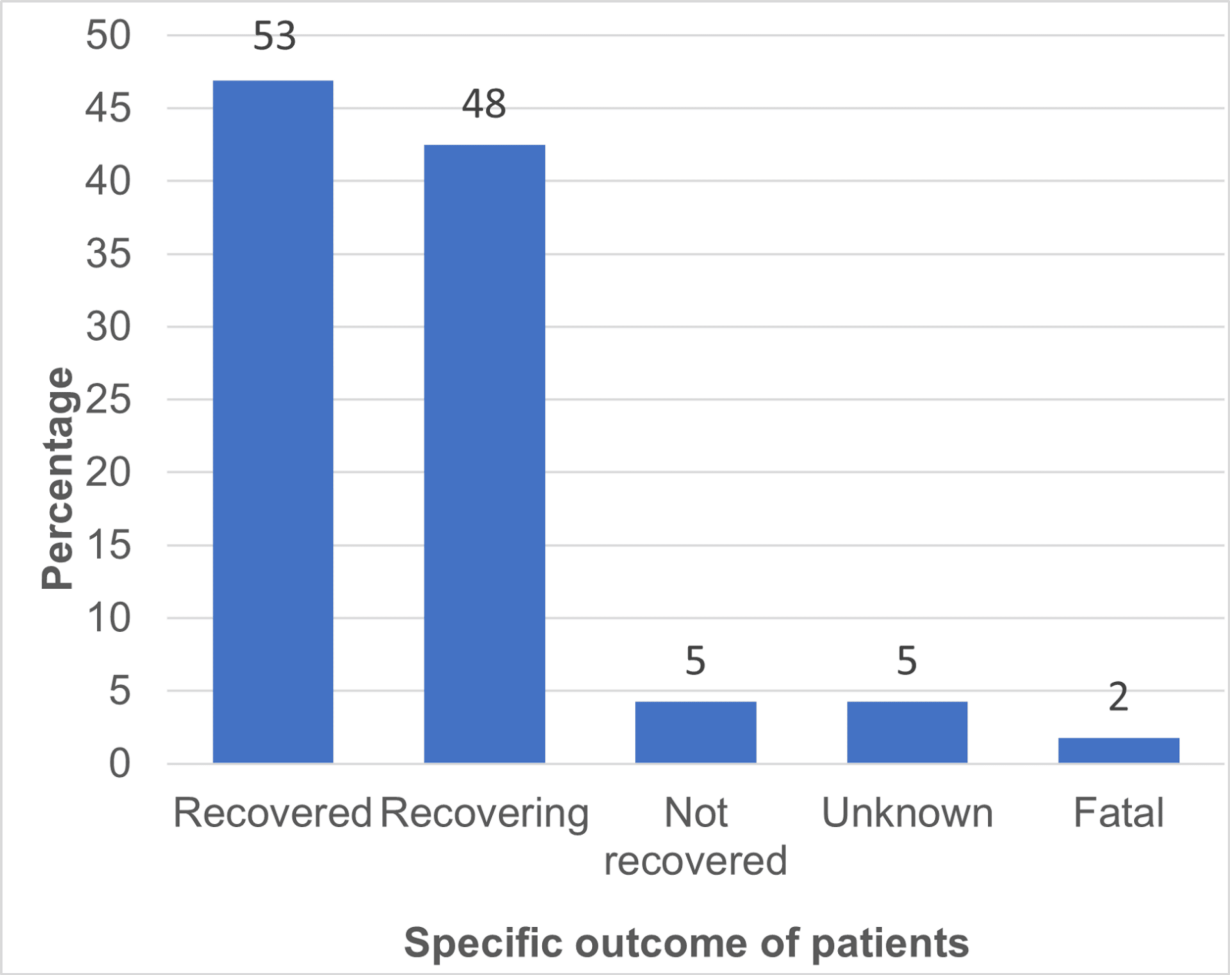
Specific outcome of the cutaneous adverse drug reactions (CADRs) (n=113)

Regarding the specific outcomes for 113 patients, 53 (46.9%) patients recovered from CADRs while 48 (42.48%) patients were still recovering at the time of discharge. The outcome was unknown and not recovered each for five (4.42%) patients. Five patients took discharge against the medical advice; hence their follow-up could not be done. There were two (1.7%) deaths seen in patients with SJS (Figure 4). 

## Discussion

Cutaneous adverse drug reactions (CADRs) are a significant concern for both patients and physicians often leading to treatment discontinuation. The development of skin eruptions is a frequent cause of this discontinuation, leading to treatment failure. CADRs contribute to patient suffering, hospitalizations, economic burdens and can occasionally be fatal. Prescribing medications to sensitized patients or those with cross-reactivity risks legal issues. A Norwegian study emphasized the importance of collecting data on CADRs including frequencies, risk factors and suspected drugs to prevent reactions. Spontaneous reporting in pharmacovigilance is crucial for detecting new or rare CADRs. However, underreporting remains a significant issue due to lack of awareness among healthcare professionals and patients [16].

In this study, 113 patients diagnosed with CADRs were analyzed. The mean age of the patients was 30.46 years ±19.67 years SD, and the median age was 29 years (range: 13.5-45 years). The majority of the patients (44.25%) were in the age group of 18-39 years, followed by 40-59 years (20.35%). This is consistent with a previous study by Karunakaran et al., which also observed a high prevalence of CADRs among patients aged 20-40 years followed by those aged 41-59 years [17]. Our study included participants ranging from three months to 86 years old mirroring the findings of Sharma et al. [18]. Notably, both studies highlight that the majority of CADRs occur within the middle-age group aligning with the substantial Indian population in this age group [18].

In our study, there was a male preponderance of 59.29% (n = 67) while 40.71% were female (n = 46) among patients with CADR, aligning with the findings of A. Modi et al. [4]. This contrasts with certain Indian studies like those by Sharma et al. [18], which reported a female preponderance in CADR cases.

We found in our study that maculopapular rash (53.98%) is the most common reaction followed by urticarial rash (9.73%) aligning with Sharma et al.'s findings [18]. Various studies have reported a frequency range of maculopapular rash from 7.7% to 60.2% [16,19-22]. SJS (8.85%) ranked as the third most common CADR, consistent with the Choon et al. study [16]. Some Indian studies highlighted fixed drug eruption as the predominant CADR [19,23]. SCARs constituted 16.81% of cases with SJS and DRESS syndrome being the most frequent among them. TEN was also observed in a few patients. SJS (8.85%) emerged as the most common severe CADR in line with several studies [16,19-23]. However, some studies identified DRESS as the primary severe CADR [5,24]. These variations in common CADRs among populations likely stem from differences in drug usage patterns and ethnic characteristics.

In this study, anti-bacterial class accounted for 42.63% of the suspected drugs causing CADR including penicillins (19.75%), sulfones (19.75%), glycopeptides (16.05%), fluoroquinolones (14.81%) and cephalosporins (9.88%). Vancomycin was the most common antibacterial responsible for CADRs. Among the antitubercular drugs (14.21%), rifampicin was the most common. Antiepileptic drugs were responsible for 11.58% of CADRs, with phenytoin being the primary cause. Other categories included antipyretics (4.74%), antifungals (4.21%) and antivirals (3.68%). These findings align with previous research by Choon et al. [16], Pudukadan et al. [19] and Nandha et al. [25]. Anti-epileptics are commonly implicated in SJS. Phenytoin was the most common drug causing SCARs consistent with findings by Noel et al. [26].

In our study, we found that in 37.17% of the cases, symptoms appeared between 2 and 24 hours after drug intake followed by >10 days being the second most common time period in 23.89% of the cases. A lag time of two to five days was observed in 21.24% of the cases, while 0-15 minutes and 6 to 10 days were seen in 7.96% and 7.08% of cases, respectively. Nandha et al. reported a majority of patients with a lag period of 2-14 days (80.2%) [25]. Early aggressive treatment within 72 hours improved prognosis, especially with prompt withdrawal of the causative drug.

The causality by WHO-UMC scale categorized 72.1% as 'possible,' 24.74% as 'probable,' and 3.16% as 'certain.' This study primarily found 'possible' causality contrasting with other studies where 'probable' causality is more common likely due to factors like combination drugs and polypharmacy complicating assessment [6,7,27,28]. Naranjo scale showed 0.53% as 'definite' and 99.47% as 'probable,' with no agreement between the two scales. We chose both scales as WHO-UMC is widely used while Naranjo minimizes variability despite its rigidity [29].

According to the modified Hartwig-Siegel severity assessment, most patients (79.65%) were classified as having moderate severity, aligning with findings from other studies [6,7,27,28]. Mild and severe groups comprised 17.7% and 2.65% of the patients, respectively. Mild ADRs mostly resolved upon stopping the suspected drug while moderate cases necessitated drug cessation along with pheniramine and hydrocortisone administration. Severe CADRs required intensive medical intervention.

In our study, according to the modified Schumock-Thornton preventability scale, 94.69% of the CADRs were deemed not preventable, possibly due to them being immunological (Type B) and unpredictable, while 5.31% of the patients had a history of similar CADRs, categorized as preventable. Factors contributing to preventable reactions included inappropriate prescribing, medication errors, self-medication, OTC drug use and neglecting allergy or CADR history [4,28,30]. Three CADRs were linked to OTC drug use (levocetirizine and herbal medicine). Although OTC availability in India is common [31], our study found only a minimal association between CADRs and OTC drug intake (three out of 113 cases).

The study assessed CADR seriousness following the ICH E2A guidelines. Of all the cases 74.34% were noted as serious correlating with the prior research by Thakkar et al [13]. Among these serious cases, 40.71% necessitated hospitalization or prolonged hospital stays due to CADR. Additionally, 30.97% of the cases had other medically significant reasons for seriousness, while 1.77% resulted in mortality and 0.88% were life-threatening. 

In this study, 46.9% of the patients recovered from CADRs while 42.28% were still recovering from CADRs at the time of discharge. Outcome was 'unknown' and 'not recovered' for five patients each. The majority of the CADR patients recovered without issues consistent with other studies [6,7,26,27]. Five patients left against medical advice hindering follow-up. Five CADR cases were due to antitubercular drugs, which were continued for multidrug-resistant (MDR) tuberculosis treatment, resulting in non-recovery of CADR. Two cases were fatal, both due to SJS. One SJS case involved phenytoin while the other had multiple suspected drugs: amoxicillin, dicyclomine, norfloxacin and metronidazole.

This study has a limitation as it only considered CADRs from the dermatology department excluding other departments of the hospital.

## Conclusions

Our study indicates that common medications prescribed in routine clinical practice may cause skin reactions; to prevent this from occurring, careful use of drugs and patients' past history of reaction is crucial. Special attention should be given while prescribing anti-bacterials. OTC drugs like levocetirizine can also trigger such responses so awareness about OTC medications among the patients has to be improved.

Patients should promptly report side effects, discontinue the drug and seek medical assistance. Implementing a comprehensive pharmacovigilance surveillance system involving the patients and healthcare professionals for baseline and follow-up documentation of patient health can significantly reduce drug-related morbidity and rationalize drug therapy, thus aiding in the prevention of CADRs.

## References

[1] World Health Organization. Quality Assurance and Safety of Medicines Team (2002). Safety of Medicines: A Guide to Detecting and Reporting Adverse Drug Reactions: Why Health Professionals Need to Take Action. https://iris.who.int/handle/10665/67378.

[2] Nayak S, Acharjya B (2008). Adverse cutaneous drug reaction. Indian J Dermatol.

[3] Bigby M (2001). Rates of cutaneous reactions to drugs. Arch Dermatol.

[4] Modi A, Desai M, Shah S, Shah B (2019). Analysis of cutaneous adverse drug reactions reported at the regional ADR Monitoring Center. Indian J Dermatol.

[5] Lee HY, Tay LK, Thirumoorthy T, Peng SM (2010). Cutaneous adverse drug reactions in hospitalised patients. Singapore Med J.

[6] Gohel D, Bhatt S, Malhotra S (2014). Evaluation of dermatological adverse drug reaction in the outpatient department of dermatology at a tertiary care hospital. Indian J Pharm Pract.

[7] Krishna J, Chitti Babu G, Goel S (2015). A prospective study of incidence and assessment of adverse cutaneous drug reactions as a part of pharmacovigilance from a rural northern Indian medical school. International Archives of Integrated Medicine.

[8] Sasidharanpillai S, Riyaz N, Khader A, Rajan U, Binitha MP, Sureshan DN (2015). Severe cutaneous adverse drug reactions: a clinicoepidemiological study. Indian J Dermatol.

[9] Li LF, Ma C (2006). Epidemiological study of severe cutaneous adverse drug reactions in a city district of China. Clin Exp Dermatol.

[10] Valeyrie-Allanore L, Sassolas B, Roujeau JC (2007). Drug-induced skin, nail and hair disorders. Drug Saf.

[11] Ajayi FO, Sun H, Perry J (2000). Adverse drug reactions: a review of relevant factors. J Clin Pharmacol.

[12] Roujeau JC, Stern RS (1994). Severe adverse cutaneous reactions to drugs. N Engl J Med.

[13] Thakkar S, Patel TK, Vahora R, Bhabhor P, Patel R (2017). Cutaneous adverse drug reactions in a tertiary care teaching hospital in India: an intensive monitoring study. Indian J Dermatol.

[14] Sharma R, Dogra D, Dogra N (2015). A study of cutaneous adverse drug reactions at a tertiary center in Jammu, India. Indian Dermatol Online J.

[15] Jha N, Alexander E, Kanish B, Badyal DK (2018). A study of cutaneous adverse drug reactions in a tertiary care center in Punjab. Indian Dermatol Online J.

[16] Choon SE, Lai NM (2012). An epidemiological and clinical analysis of cutaneous adverse drug reactions seen in a tertiary hospital in Johor, Malaysia. Indian J Dermatol Venereol Leprol.

[17] Karunakaran P, Kakkanatt A, Bai J (2020). Cutaneous adverse drug reactions (CADRs) at a quaternary care hospital in South India: focus on reaction time and treatment cost. Indian J Pharm Pract.

[18] Sharma S, Jayakumar D, Palappallil DS (2019). Pharmacovigilance of cutaneous adverse drug reactions among patients attending dermatology department at a tertiary care hospital. Indian Dermatol Online J.

[19] Pudukadan D, Thappa DM (2004). Adverse cutaneous drug reactions: clinical pattern and causative agents in a tertiary care center in South India. Indian J Dermatol Venereol Leprol.

[20] Patel TK, Thakkar SH, Sharma DC (2014). Cutaneous adverse drug reactions in Indian population: a systematic review. Indian Dermatol Online J.

[21] Mitra J, Hamide A, Hassan M (2009). Prevalence of cutaneous drug eruption in hospitalized patients: a report from Sina Hospital of Tabriz. Iran J Dermatol.

[22] Anjaneyan G, Gupta R, Vora RV (2013). Clinical study of adverse cutaneous drug reactions at a rural based tertiary care centre in Gujarat. Natl J Physiol Pharm Pharmacol.

[23] Radhika MS, Mayur SS, Kop P (2013). Pattern of cutaneous adverse drug reactions due to the use of fixed dose drug combinations. International Journal of Basic and Clinical Pharmacology.

[24] Akpinar F, Dervis E (2012). Drug eruptions: an 8-year study including 106 inpatients at a dermatology clinic in Turkey. Indian J Dermatol.

[25] Nandha R, Gupta A, Hashmi A (2011). Cutaneous adverse drug reactions in a tertiary care teaching hospital: a North Indian perspective. Int J Appl Basic Med Res.

[26] Noel MV, Sushma M, Guido S (2004). Cutaneous adverse drug reactions in hospitalized patients in a tertiary care center. Indian J Pharmacol.

[27] Shah SP, Desai MK, Dikshit RK (2011). Analysis of cutaneous adverse drug reactions at a tertiary care hospital - a prospective study. Trop J Pharm Res.

[28] Padmavathi S, Manimekalai K, Ambujam S (2013). Causality, severity and preventability assessment of adverse cutaneous drug reaction: a prospective observational study in a tertiary care hospital. J Clin Diagn Res.

[29] Shukla AK, Jhaj R, Misra S, Ahmed SN, Nanda M, Chaudhary D (2021). Agreement between WHO-UMC causality scale and the Naranjo algorithm for causality assessment of adverse drug reactions. J Family Med Prim Care.

[30] Ghosh S, Acharya L, Rao P (2006). Study and evaluation of the various cutaneous adverse drug reactions in Kasturba Hospital, Manipal. Indian J Pharm Sci.

[31] Bhanwra S (2013). A study of non-prescription usage of antibiotics in the upper respiratory tract infections in the urban population. J Pharmacol Pharmacother.

